# Vitamin D Deficiency as a Risk Factor for Onset and Recurrence of Sudden Sensorineural Hearing Loss: A Prospective Cohort Study With Age‐Specific Analysis

**DOI:** 10.1002/fsn3.71383

**Published:** 2026-01-14

**Authors:** Qi Hui, Tu Xiang, Xiong Qinglan, Yang Shuowei, Chi Mengshi, Xiong Qin, Liu Hongyin, Yang Juntao, Fan Yunping

**Affiliations:** ^1^ Department of Otolaryngology, The Seventh Afffliated Hospital Sun Yat‐Sen University Shenzhen China; ^2^ Otorhinolaryngology Hospital, The First Affiliated Hospital Sun Yat‐Sen University Guangzhou China; ^3^ School of Medicine Sun Yat‐Sen University Shenzhen China

**Keywords:** hearing recovery, recurrence, sudden sensorineural hearing loss (SSNHL), vitamin D (Vit D), younger adults

## Abstract

The link between Vitamin D (Vit D) deficiency and sudden sensorineural hearing loss (SSNHL), especially regarding recurrence and age‐related disparities, remains unclear. Recurrence and age‐specific risks of SSNHL are under addressed, limiting preventive strategies. This study investigated if Vit D deficiency increases SSNHL onset and recurrence risks, with a focus on age‐specific associations. In this prospective cohort study, 80 adult SSNHL patients and 60 matched controls were enrolled. All patients received standard therapy. Baseline serum 25(OH)D was measured, Vit D deficiency or insufficient patients were randomized in a single‐blind manner to receive 3‐month Vit D supplementation. We monitored Vit D levels, hearing recovery (pure‐tone audiometry at 10 days and 3 months), recurrence, and associated symptoms. Vit D deficiency was more prevalent in SSNHL patients than controls (38.8% vs. 10.0%, *p* = 0.000). Recurrent SSNHL patients had significantly lower Vit D levels (18.4 ± 4.1 ng/mL) than first‐onset patients (24.9 ± 9.7 ng/mL). The highest deficiency rate (60.0%) was in patients under 30 years. Multivariate analysis identified audiometric subtype, not Vit D status, as an independent outcome predictor. Vit D supplementation did not significantly improve recovery rates (75.7% vs. 68.2%) or alleviate symptoms. Vit D deficiency is a significant, modifiable risk factor for SSNHL onset and recurrence, with the strongest association in patients under 30 years old. However, short‐term Vit D supplementation initiated after SSNHL diagnosis failed to improve clinical outcomes, underscoring that long‐term maintenance of adequate Vit D levels, rather than acute post‐symptom intervention, may be critical for reducing SSNHL risk in high‐risk populations.

## Graphical abstract

Pure‐tone audiometry (0–120 dB HL) identified patients with sudden sensorineural hearing loss (SSNHL). Serum 25‐hydroxyvitamin D levels revealed deficient (< 20 ng/mL), insufficient (20–30 ng/mL), or sufficient (≥ 30 ng/mL) status. Novel findings demonstrate low vitamin D correlates with higher SSNHL onset/recurrence, particularly elevating risk in younger populations (< 30 years). Preventive vitamin D supplementation is proposed as a strategic intervention for high‐risk groups, emphasizing its potential role in mitigating auditory morbidity. HL, hearing level; SSNHL, sudden sensorineural hearing loss; VD, Vitamin D.

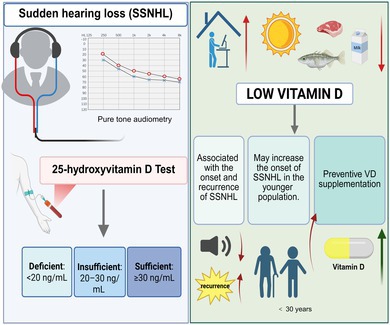

## 1 Introduction

Sudden sensorineural hearing loss (SSNHL) is defined as a rapid decline in auditory function (≥ 30 dB hearing loss across three consecutive frequencies within 72 h), which poses a significant public health challenge due to its potential to cause permanent hearing impairment and reduce quality of life. The global incidence of SSNHL ranges from 5 to 400 per 100,000 individuals annually, with a rising trend worldwide, highlighting the urgency of identifying modifiable risk factors for prevention and intervention (Yang et al. 2024).

For decades, potential etiologies of SSNHL have included vascular insufficiency, autoimmune responses, and viral infections, but a definitive cause remains elusive (Arunthanachaikul et al. 2025; Li, Han, et al. 2024; Yamada et al. 2022). In recent years, accumulating evidence has linked Vitamin D (Vit D) deficiency to auditory impairment, particularly SSNHL (Bigman 2022; Chen et al. 2024; Ghazavi et al. 2020; Karaer and Akalın 2020; Lee et al. 2024; Paprocki et al. 2021; Szeto et al. 2021). This association is biologically plausible: Vit D regulates cochlear calcium homeostasis, mitigates oxidative stress via antioxidant enzyme activation, and modulates inflammatory responses, while Vit D deficiency contributes to inner ear damage (Bigman 2022; Paprocki et al. 2021; Szeto et al. 2021; Zhang et al. 2022). However, three key gaps persist in current research: (1) Most studies have focused on overall SSNHL populations, with no clear analysis of age‐specific associations, whether Vit D deficiency has a stronger impact on younger adults; (2) No studies have explored the relationship between Vit D levels and SSNHL recurrence, leaving the role of Vit D in disease progression unclear; (3) Despite widespread interest in Vit D supplementation, there is no evidence to confirm whether supplementation improves hearing recovery in deficient SSNHL patients. These unresolved gaps limit clinical guidance for SSNHL prevention and treatment.

To address these gaps, we adopted a prospective cohort study design, which minimizes selection bias by enrolling participants at the time of SSNHL diagnosis (rather than relying on historical data) and enables direct comparison of Vit D deficiency prevalence between SSNHL patients and the general population via age‐ and sex‐matched controls, thereby enhancing the validity of causal inferences (Luo et al. 2018). The primary objective of this study was to: (1) verify whether Vit D deficiency is an independent risk factor for SSNHL, particularly in younger adults and patients with recurrent episodes; (2) evaluate whether adjunctive Vit D supplementation (in deficient patients) improves hearing recovery compared to standard care alone.

## 2 Methods and Materials

### 2.1 Study Design and Participants

This study adopted a prospective cohort design, integrating cross‐sectional analysis and longitudinal follow‐up with clearly defined objectives for each phase. The cross‐sectional phase focused on comparing serum 25(OH)D_3_ levels between newly diagnosed sudden sensorineural hearing loss (SSNHL) patients and age‐ and sex‐matched healthy controls, aiming to explore whether Vit D deficiency constitutes an independent risk factor for SSNHL. The longitudinal phase involved 3‐month follow‐up of SSNHL patients to analyze the association between baseline Vit D status and disease recurrence, while evaluating the efficacy of Vit D supplementation on hearing recovery.

Sample size was calculated using G.Power 3.1 software based on the primary outcome: the difference in Vit D deficiency prevalence between SSNHL patients and controls. Referring to a previous study by Ghazavi et al. (2020), the prevalence of 25(OH)D_3_ < 30 ng/mL in the SSNHL patients was estimated at 76.5%. With a significance level (α) of 0.05, power (1‐β) of 0.80, and a case–control ratio of 1:1, the minimum sample size required was 114 participants (57 SSNHL patients and 57 controls). Considering a potential dropout rate of 20% during the 3‐month follow‐up, we expanded the sample size to 144 participants (72 SSNHL patients and 72 controls) to ensure sufficient statistical power for both cross‐sectional and longitudinal analyses. The actual number of participants enrolled in this study was 80 SSNHL patients and 60 healthy controls; the sample size still met the required statistical power.

SSNHL patients were consecutively recruited from the Department of Otolaryngology Surgery at the Seventh Affiliated Hospital of Sun Yat‐sen University between January 2022 and December 2024. Inclusion criteria were defined as: (1) age ≥ 18 years; (2) diagnosis of idiopathic SSNHL confirmed by pure‐tone audiometry (PTA), characterized by a ≥ 30 dB hearing loss across three consecutive frequencies within 72 h; (3) no prior history of SSNHL, chronic otological disorders (e.g., Meniere's disease, otosclerosis), or permanent hearing impairment; (4) ability to comply with follow‐up procedures and provide informed consent. Exclusion criteria included: (1) pregnancy, lactation, or plans for pregnancy during the study period; (2) concurrent participation in other clinical trials; (3) declination to undergo blood sampling or serial audiometric assessments.

Healthy controls were recruited from the hospital's preventive medicine center during the same period, matched to SSNHL patients by age (±3 years) and sex. Inclusion criteria for controls were: (1) normal hearing function (PTA thresholds ≤ 25 dB HL at 250–8000 Hz); (2) no history of otological diseases, chronic systemic disorders, or Vit D supplementation within 3 months; (3) no family history of hereditary hearing loss. Controls underwent the same baseline assessments (audiometry and biochemical testing) as SSNHL patients to confirm eligibility.

This study was approved by the Ethics Committee of the Seventh Affiliated Hospital of Sun Yat‐sen University (Protocol Code: KY AF/SC‐07/01.0) and conducted in strict adherence to the Declaration of Helsinki. Prior to the study initiation, all participants were fully informed of the purpose of the study, procedures, potential risks, and benefits. Written informed consent was obtained after participants had a clear understanding of the study, documenting their right to withdraw from the study at any time without prejudice to subsequent clinical treatment.

### 2.2 Treatment Protocol

All SSNHL patients received standardized first‐line treatment for SSNHL, consisting of oral methylprednisolone (1 mg/kg/day, maximum 60 mg/day) for 7 days, followed by a tapering regimen (reducing by 10 mg every 3 days until discontinuation on Day 21) and oral *Ginkgo biloba* extract (120 mg twice daily for 30 days) to improve cochlear microcirculation. Vit D deficiency or insufficient patients (25(OH)D_3_ < 30 ng/mL (Holick et al. 2011)) were randomized in a single‐blind fashion to either a Vit D supplementation group or a non‐supplementation group. Patients in the supplementation group received oral vitamin D2 (800 IU/day) and D3 (250 IU/day) for 3 months, a dose recommended for deficiency correction in adults (Holick et al. 2011). To ensure compliance, monthly telephone interviews were conducted to monitor adherence and document adverse events (e.g., gastrointestinal discomfort, hypercalcemia symptoms). Participants were advised to avoid additional Vit D supplements, high‐dose Vit D‐fortified foods (e.g., fortified milk, fish oil supplements), and excessive sunlight exposure (≥ 2 h/day) throughout the study period.

### 2.3 Audiologic Evaluation

Auditory function was evaluated using pure‐tone audiometry (PTA) at three standardized time points: baseline (within 24 h after enrollment), 10 days post‐treatment, and 3 months post‐enrollment. According to the World Health Organization (WHO) criteria (Braffett et al. 2019; Olusanya et al. 2019), the severity of hearing loss was classified as: normal hearing (PTA < 20 dB HL), mild impairment (20 to < 35 dB HL), moderate impairment (35 to < 50 dB HL), moderate–severe impairment (50 to < 65 dB HL), severe impairment (65 to < 80 dB HL), profound impairment (80 to < 95 dB HL), and total deafness (≥ 95 dB HL). Patients were further categorized into audiometric subtypes based on the affected frequency ranges: low‐frequency SSNHL (LF‐SSNHL, 125–1000 Hz), high‐frequency SSNHL (HF‐SSNHL, 2000–8000 Hz), all‐frequency SSNHL (AF‐SSNHL, 250–8000 Hz), and total deafness SSNHL (TD‐SSNHL, threshold ≥ 81 dB across all frequencies). Additionally, patients were classified as primary or recurrent SSNHL based on the number of episodes. Hearing recovery was defined as a ≥ 15 dB improvement in hearing threshold at affected frequencies on PTA; unimproved hearing was defined as an improvement of < 15 dB. SSNHL recurrence was strictly defined as the new onset of sudden hearing loss occurring at least 3 months after initial hearing recovery (Ko et al. 2024). All recurrent cases were confirmed by rechecking PTA within 48 h of symptom onset to rule out transient hearing fluctuations.

### 2.4 Biochemical Measurements and Symptom Assessment

Biochemical analysis was performed on baseline serum samples (5 mL) using chemiluminescent immunoassay to quantify 25(OH)D and parathyroid hormone (PTH) levels. Vit D status was defined as deficient (< 20 ng/mL), insufficient (20–30 ng/mL), or sufficient (≥ 30 ng/mL). Biochemical control group data was obtained from routine health examination records. We evaluated accompanying symptoms including tinnitus, vertigo, and aural fullness using visual analog scales (VAS), considering symptom improvement as a reduction in VAS scores during follow‐up assessments.

### 2.5 Statistical Analysis

Data analysis was performed using SPSS 29.0 (IBM, Armonk, NY, USA). The normality of continuous variables was first determined by the Shapiro–Wilk test. Normally distributed variables were expressed as mean ± standard deviation (mean ± SD), while non‐normally distributed variables were reported as median (interquartile range, IQR); categorical variables were presented as frequency (percentage).

For inter‐group comparisons: Student's *t*‐test was used for normally distributed continuous variables, the Mann–Whitney *U* test for non‐normally distributed continuous variables, and the Chi‐square test or Fisher's exact test for categorical variables as appropriate. Multivariate logistic regression analysis was used to explore the association between predictor variables (Vit D status, age, audiometric subtype, symptom characteristics) and outcome variables (hearing recovery, recurrence); this model adjusted for potential confounding factors (age, sex, severity of hearing loss) to identify independent influencing factors. Effect sizes were expressed as odds ratios (OR) with 95% confidence intervals (95% CI) to reflect clinical significance. A two‐tailed test was used for all analyses, with *p* < 0.05 considered statistically significant.

## 3 Results

### 3.1 Participant Characteristics

This study enrolled a total of 140 participants, including 80 patients with sudden sensorineural hearing loss (SSNHL) and 60 age‐ and sex‐matched healthy controls. The baseline demographic characteristics of the two groups were comparable, with no statistically significant differences in sex distribution (55.0% males in the SSNHL group vs. 53.3% males in the control group, *p* = 0.845) or age (mean ± standard deviation [SD]: 41.2 ± 11.9 years in the SSNHL group vs. 44.0 ± 14.8 years in the control group, *p* = 0.230). As expected, the mean pure‐tone audiometry (PTA) threshold of the SSNHL group was significantly higher than that of the control group (68.4 ± 26.4 dB vs. 11.6 ± 5.8 dB, *p* = 0.000) (Table 1).

**TABLE 1:** Baseline characteristics of participants.

Characteristics	SSNHL (*n* = 80)	Control (*n* = 60)	*p*
Sex, *n* (%)			
Male	44 (55.0)	32 (53.3)	0.845
Female	36 (45.0)	28 (46.7)	
Age, mean (SD), years	41.2 (11.9)	44.0 (14.8)	0.230
PTA, mean (SD), dB	68.4 (26.4)	11.6 (5.8)	**0.000**

*Note:* Sex and age are baseline demographic variables for group comparability verification, not core outcome variables of the study. Bold values indicate statistical significance (*p* < 0.05).

Abbreviations: *n*, number; PTA, pure‐tone audiometry; SD, standard deviation; SSNHL, sudden sensorineural hearing loss.

### 3.2 Factors Associated With Hearing Recovery

Hearing recovery was defined as a ≥ 15 dB improvement in the PTA threshold at the affected frequencies. Among the 80 SSNHL patients, 57 (71.3%) achieved hearing recovery, while 23 (28.8%) showed no significant improvement. Multivariate logistic regression analysis showed that treatment PTA threshold and SSNHL frequency subtype were independent predictors of hearing recovery. Specifically: Patients with low‐frequency SSNHL (LF‐SSNHL) had a significantly higher recovery rate than those with high‐frequency SSNHL (HF‐SSNHL) (45.6% vs. 8.7%, adjusted odds ratio [aOR] = 36.157, 95% confidence interval [CI]: 1.380–240.517, *p* = 0.031). Patients with total deafness SSNHL (TD‐SSNHL) had a poorer prognosis, with an unrecovery rate of 43.5% (vs. 8.7% in the LF‐SSNHL group) (Tables 2 and 3). Univariate analysis showed a trend toward better recovery in younger patients (< 30 years) (odds ratio [OR] = 7.90, 95% CI: 0.970–64.174, *p* = 0.053), but this association was no longer statistically significant after adjusting for confounding factors (*p* = 0.218). No statistically significant associations were found between hearing recovery and other factors, including sex, comorbidities, baseline severity of hearing loss, or Vit D status (Table 3).

**TABLE 2:** Demographic data and clinical characteristics of SSNHL patients stratified by hearing recovery status.

Characteristic	Improved (*n* = 57)	Unimproved (*n* = 23)
Sex, *n* (%)		
Male	28 (49.1)	16 (69.6)
Female	29 (50.9)	7 (30.4)
Affected ear, *n* (%)		
Left	33 (57.9)	16 (69.6)
Right	24 (42.1)	7 (30.4)
Age, *n* (%)		
Total age, mean (SD)	39.5 (12.3)	45.4 (10.0)
< 30	14 (24.6)	1 (4.3)
30–60	37 (64.9)	18 (78.3)
≥ 60	6 (10.5)	4 (17.4)
Accompanying symptoms, *n* (%)		
Tinnitus	51 (89.5)	19 (83.6)
Vertigo	15 (26.3)	8 (34.8)
Ear fullness	33 (57.9)	12 (52.3)
Degree of hearing loss, *n* (%)		
LF‐SSNHL	26 (45.6)	2 (8.7)
HF‐SSNHL	2 (3.6)	2 (8.7)
AF‐SSNHL	16 (28.1)	9 (39.1)
TD‐SSNHL	13 (22.8)	10 (43.5)
Type of SSNHL, *n* (%)		
Mild	7 (12.3)	1 (4.3)
Moderate	16 (28.1)	5 (21.7)
Moderately severe	9 (15.8)	3 (13.0)
Severe	10 (17.5)	3 (13.0)
Profound	5 (8.8)	3 (13.0)
Deafness	10 (17.5)	8 (34.8)
Systemic disease, *n* (%)		
Diabetes	5 (8.7)	1 (4.3)
Hypertension	5 (8.7)	2 (8.7)
25 (OH)D, *n* (%)		
Sufficient	15 (26.3)	6 (26.1)
Insufficient	20 (35.1)	8 (34.8)
Deficient	22 (38.6)	9 (39.1)

*Note:* 25(OH)D status was defined as sufficient (≥ 30 ng/mL), insufficient (20–30 ng/mL), and deficient (< 20 ng/mL).

Abbreviations: 25(OH)D, 25‐hydroxyvitamin D; AF‐SSNHL, all‐frequency SSNHL; HF‐SSNHL, high‐frequency SSNHL; LF‐SSNHL, low‐frequency SSNHL; *n*, number; SD, standard deviation; TD‐SSNHL, total deafness SSNHL.

**TABLE 3:** Univariate and multivariate logistic regression analyses of treatment outcome.

Parameter	Univariate analysis			Multivariate analysis		
	OR	95% CI	*P* value	aOR	95% CI	*P* value
Treatment PTA	1.049	1.027–1.072	**0.000**	1.405	1.140–1.731	**0.001**
Sex						
Male	1					
Female	0.39	0.139–1.103	0.076			
Affected ear						
Left	1					
Right	0.56	0.201–1.592	0.280			
Age, years						
< 30	1			1		
30–60	7.90	0.970–64.174	0.053	4.841	0.394–59.547	0.218
≥ 60	15.00	0.896–251.056	0.060	7.898	0.162–384.921	0.297
Accompanying symptom						
Tinnitus	2.69	0.609–11.823	0.192			
Vertigo	0.70	0.248–1.996	0.508			
Ear fullness	1.38	0.516–3.664	0.524			
Type of SSNHL						
LF‐SSNHL	1			1		
HF‐SSNHL	12.00	1.053–136.794	**0.045**	36.157	1.380–240.517	**0.031**
AF‐SSNHL	6.75	1.286–35.416	**0.024**	6.103	0.983–37.893	0.053
TD‐SSNHL	9.23	1.753–48.618	**0.009**	10.239	0.260–403.152	0.215
Degree of hearing loss						
Mild	1					
Moderate	1.56	0.146–16.719	0.712			
Moderately severe	1.66	0.135–20.578	0.690			
Severe	1.50	0.123–18.363	0.751			
Profound	3.00	0.227–39.608	0.404			
Deafness	4.00	0.385–41.511	0.246			
Systemic disease						
Diabetes	2.20	0.243–19.951	0.483			
Hypertension	1.05	0.189–5.847	0.956			
25 (OH)D						
Sufficient	1					
Insufficient	1.09	0.348–3.390	0.886			
Deficient	0.98	0.288–3.325	0.971			

*Note:* Variables with *p* > 0.10 in univariate analysis were excluded from multivariate modeling. Bold values indicate statistical significance (*p* < 0.05). Male was set as the reference group for sex stratification per conventional clinical epidemiological practice.

Abbreviations: aOR, adjusted odds ratio; CI, confidence interval; OR, odds ratio; other abbreviations as per Table 2; PTA, pure‐tone audiometry.

### 3.3 Vitamin D Status and Biochemical Profiles

Before treatment, the prevalence of Vit D deficiency (defined as 25‐hydroxyvitamin D [25(OH)D] < 20 ng/mL) in patients with SSNHL was 38.8%, which was significantly higher than that in age‐ and sex‐matched healthy controls (10.0%, *p* = 0.000). Patients with recurrent SSNHL (*n* = 10) exhibited markedly lower 25(OH)D levels compared to those with first‐onset SSNHL (18.4 ± 4.1 ng/mL vs. 24.9 ± 9.7 ng/mL, *p* = 0.000) and healthy controls (18.4 ± 4.1 ng/mL vs. 32.6 ± 11.0 ng/mL, *p* = 0.000). Additionally, the Vit D deficiency rate in the recurrent group (80.0%) was substantially higher than that in the first‐onset group (32.5%, *p* = 0.000).

Age‐stratified analysis revealed that patients under 30 years old had the highest prevalence of Vit D deficiency (60.0%), followed by those aged 30–60 years (33.9%), while no Vit D deficiency was observed in patients over 60 years old. Parathyroid hormone (PTH) levels were inversely correlated with Vit D status: the mean PTH level in patients with recurrent SSNHL (67.9 ± 19.7 ng/mL) was significantly higher than that in healthy controls (47.9 ± 13.1 ng/mL, *p* = 0.000). These associations remained stable after adjusting for major confounding factors via age and sex matching. No statistically significant correlation was found between serum Vit D levels and the baseline severity of hearing loss (*p* = 0.496) (Table 4).

**TABLE 4:** 25(OH)VD/PTH levels among the study groups before treatment.

Groups	25(OH)VD means (SD)	PTH means (SD)	Category of 25(OH)VD (%)		
			Sufficient	Insufficient	Deficient
SSNHL (*n* = 80)	24.1 (9.5)	57.0 (23.3)	21 (26.2)	28 (35.0)	31 (38.8)
Primary SSNHL (*n* = 70)	24.9 (9.7)	53.4 (22.8)	21 (28.0)	28 (37.3)	26 (32.5)
Recurrent SSNHL (*n* = 10)	18.4 (4.1)	67.9 (19.7)	0 (0.0)	2 (20.0)	8 (80.0)
Control (*n* = 60)	32.6 (11.0)	47.9 (13.1)	35 (58.3)	19 (31.7)	6 (10.0)
*p*	**0.012**	**0.042**	**0.000**		
SSNHL (*n* = 80)					
Age, years					
< 30	18.2 (5.7)	53.9 (25.9)	0 (0.0)	6 (40.0)	9 (60.0)
30–60	24.8 (9.3)	59.6 (22.2)	18 (30.5)	21 (35.6)	20 (33.9)
≥ 60	35.4 (12.8)	30.0 (6.6)	3 (75.0)	1 (25.0)	0 (0.0)
*p*	**0.010**	**0.039**	**0.007**		
Type Of SSNHL					
LF‐SSNHL	22.1 (8.5)	58.5 (24.9)	5 (19.2)	9 (34.6)	12 (46.2)
HF‐SSNHL	22.1 (9.2)	58.7 (30.0)	4 (66.7)	0 (0.0)	2 (33.3)
AF‐SSNHL	27.4 (11.4)	55.7 (20.8)	9 (36.0)	9 (36.0)	7 (28.0)
TD‐SSNHL	23.0 (8.0)	56.3 (24.2)	5 (22.7)	8 (36.4)	9 (40.9)
*p*	0.200	0.973	0.256		
Degree of Hearing loss					
Mild	30.2 (9.0)	65.6 (30.6)	3 (50.0)	2 (33.3)	1 (16.7)
Moderate	24.1 (11.6)	54.8 (22.6)	6 (28.6)	6 (28.6)	9 (42.8)
Moderately severe	23.7 (5.5)	50.7 (23.9)	2 (14.3)	8 (57.1)	4 (28.6)
Severe	24.1 (7.9)	61.1 (29.2)	4 (30.7)	5 (38.4)	4 (30.7)
Profound	23.4 (9.3)	54.8 (17.9)	2 (25.0)	3 (37.5)	3 (37.5)
Deafness	23.1 (8.1)	56.6 (22.4)	5 (27.8)	5 (27.8)	8 (44.4)
*p*	0.496	0.373	0.070		

*Note:* *p*‐values were calculated via one‐way ANOVA (continuous variables: 25(OH)D, PTH) and Chi‐square test (categorical variables: VD deficiency rate). Values with bold indicate statistical significance (*p* < 0.05).

Abbreviations: 25(OH)D, 25‐hydroxyvitamin D; *n*, number; PTH, parathyroid hormone; SD, standard deviation; SSNHL, sudden sensorineural hearing loss; VD, vitamin D.

### 3.4 Effect of Vitamin D Supplementation

There were no statistically significant differences in hearing recovery rates among the Vit D‐supplemented (75.7%), non‐supplemented (68.2%), and Vit D‐sufficient (70.0%) groups (*p* = 0.715). Similarly, no statistically significant differences were observed in the improvement of accompanying symptoms (tinnitus, vertigo, ear fullness) among the three groups (all *p* > 0.05) (Table 5).

**TABLE 5:** Evaluation of clinical symptoms under different conditions of VD supplementation.

Clinical symptoms		VD supplement *n* (%)	No VD supplement *n* (%)	VD sufficiency *n* (%)	*p*
PTA (*n* = 80)	Improved	28 (75.7)	15 (68.2)	14 (70.0)	0.715
	Unimproved	9 (24.3)	7 (31.8)	7 (30.0)	
Tinnitus (*n* = 70)	Improved	26 (86.7)	18 (81.8)	16 (88.9)	0.801
	Unimproved	4 (13.3)	4 (18.2)	2 (11.1)	
Vertigo (*n* = 23)	Improved	8 (80.0)	6 (85.7)	7 (87.5)	0.902
	Unimproved	2 (20.0)	1 (14.3)	1 (12.5)	
Ear fullness (*n* = 45)	Improved	15 (88.2)	14 (87.5)	11 (91.7)	0.936
	Unimproved	2 (11.8)	2 (12.5)	1 (8.3)	

*Note:* VD supplement group included patients with VD deficiency/insufficiency who received 3‐month supplementation (800 IU/day D_2_ + 250 IU/day D_3_); VD sufficient group had baseline 25(OH)D ≥ 30 ng/mL without supplementation. No values reached statistical significance (all *p* > 0.05).

Abbreviations: *n*, number; PTA, pure‐tone audiometry; VD, vitamin D.

## 4 Discussion

The rising global incidence of sudden sensorineural hearing loss (SSNHL) underscores the urgency of identifying modifiable risk factors (Chandrasekhar et al. 2019; Editorial Board of Chinese Journal of Otorhinolaryngology Head and Neck Surgery; Society of Otorhinolaryngology Head and Neck Surgery, Chinese Medical Association 2015). Our findings identify low Vit D status as a preventable nutritional contributor to SSNHL, akin to its established roles in cardiovascular and autoimmune diseases (Gaudet et al. 2022; Li, Huang, et al. 2024; Pop et al. 2022).

### 4.1 Association Between Vitamin D Deficiency and SSNHL

This study found that the serum 25(OH)D level in SSNHL patients (24.1 ± 9.5 ng/mL) was significantly lower than that in healthy controls (32.6 ± 11.0 ng/mL, *p* = 0.012), with 73.7% of patients presenting with Vit D insufficiency or deficiency (< 30 ng/mL)‐ a prevalence 1.8 times higher than that in the control population (41.7%). This aligns with previous research conclusions in pediatric, female, and elderly populations (Karaer and Akalın 2020; Kose Celebi et al. 2023; Mehta et al. 2020; Zandi et al. 2023; Zheng et al. 2023). From a biological mechanism perspective, this association is biologically plausible: Vit D maintains the stability of endolymphatic potential through regulating cochlear calcium‐binding proteins, ensuring the mechanotransduction function of hair cells (Paprocki et al. 2021; Zou et al. 2008) [7, 27]; meanwhile, Vit D can activate antioxidant enzymes such as glutathione peroxidase to reduce ischemic or noise‐induced cochlear oxidative damage (Paprocki et al. 2021), and inhibit pro‐inflammatory cytokines like TNF‐α and IL‐6 to reduce immune‐mediated inner ear damage (Licata et al. 2025). Animal experiments further confirm this relationship, as Vit D receptor (VDR)‐knockout mice exhibit accelerated degeneration of spiral ganglion neurons (Zou et al. 2008).

### 4.2 Relationship Between Vitamin D Status and SSNHL Recurrence

This study is the first to focus on the association between Vit D levels and SSNHL recurrence. It found that patients with recurrent SSNHL had significantly lower 25(OH)D levels (18.4 ± 4.1 ng/mL) than those with first‐onset SSNHL (24.9 ± 9.7 ng/mL, *p* < 0.001), and the parathyroid hormone (PTH) level in recurrent patients (67.9 ± 19.7 ng/mL) was significantly higher than that in controls (47.9 ± 13.1 ng/mL, *p* = 0.000). This suggests that disorders of the Vit D‐PTH axis may be involved in the recurrence of SSNHL.

This phenomenon can be explained by the “threshold effect”: when 25(OH)D < 20 ng/mL, the regulatory role of Vit D in immunity and metabolism is lost, similar to the pattern of “Vit D deficiency triggering disease flares” in autoimmune diseases (Charoenngam and Holick 2020). Chronic Vit D insufficiency may exacerbate cochlear microvascular dysfunction and neuronal apoptosis, reducing the inner ear's tolerance to subsequent damage and ultimately increasing the risk of recurrence. This result has implications for clinical management: for patients with a history of SSNHL, monitoring and maintaining a 25(OH)D level > 30 ng/mL may be key to reducing the recurrence risk, rather than only focusing on Vit D levels during acute episodes.

### 4.3 Youth Vulnerability: A Silent Epidemic of Low Vitamin D Status

Notably, our age‐stratified analysis revealed a striking trend: younger patients (< 30 years) exhibited the lowest serum 25(OH)D levels (18.2 ± 5.7 ng/mL), significantly lower than those in patients aged 30–60 years (24.8 ± 9.3 ng/mL) and > 60 years (35.4 ± 12.8 ng/mL; *p* = 0.000). Specifically, 60% of young SSNHL patients met the criteria for severe Vit D deficiency (< 20 ng/mL), a finding that coincides with emerging epidemiological evidence indicating a growing trend of younger age at SSNHL onset (Sun et al. 2024)—recurrent SSNHL has also been increasingly reported in this demographic (Ko et al. 2024), prompting reconsideration of the clinical relevance of Vit D supplementation in young adults. This age‐related Vit D deficiency pattern is likely attributed to modern lifestyle factors, including increased indoor occupations, frequent sunscreen use, and high consumption of processed foods—all of which reduce sun exposure (the primary source of endogenous Vit D synthesis) and dietary Vit D intake. We acknowledge that the small sample size of the young adult subgroup may limit the statistical power of our findings, which should be interpreted with caution. Nevertheless, considering Vit D's well‐documented roles in cochlear calcium homeostasis, neural plasticity, and antioxidant defense, early‐life Vit D deficiency may confer long‐term susceptibility to auditory dysfunction. These observations highlight the need for targeted Vit D screening in young adults presenting with audiovestibular symptoms, even in the absence of traditional risk factors. Clinically, young individuals with prolonged indoor work patterns may benefit from proactive interventions such as Vit D supplementation or increased outdoor light exposure to maintain adequate serum Vit D levels, potentially mitigating the risk of SSNHL onset or recurrence.

### 4.4 Influencing Factors of Hearing Recovery and Efficacy of Vitamin D Supplementation

Multivariate analysis in this study showed that only the audiometric subtype of SSNHL was significantly associated with hearing recovery: patients with LF‐SSNHL had the most obvious recovery advantage (aOR = 36.157, *p* = 0.031), while patients with all‐frequency SSNHL (AF‐SSNHL) and total deafness SSNHL (TD‐SSNHL) had lower recovery rates (the non‐recovery rate of TD‐SSNHL was 45.3%). This is consistent with the consensus in domestic and international SSNHL diagnosis and treatment guidelines that “the low‐frequency subtype has the best prognosis” (Editorial Board of Chinese Journal of Otorhinolaryngology Head and Neck Surgery; Society of Otorhinolaryngology Head and Neck Surgery, Chinese Medical Association 2015). Notably, although Vit D deficiency is associated with SSNHL onset, there was no significant association between Vit D status (deficient/insufficient/sufficient) and hearing recovery (*p* > 0.05), and short‐term supplementation also failed to improve the recovery rate. This seemingly “contradictory” result reflects the role of Vit D in SSNHL: it acts as a pathogenic risk factor rather than a therapeutic intervention target for acute disease.

From the perspective of the intervention protocol, the ineffectiveness of short‐term supplementation may be attributed to two factors: First, the supplementation dose (a total of 1050 IU per day) and duration (3 months) used in this study may be insufficient. Correcting severe Vit D deficiency typically requires a higher dose (e.g., 4000 IU per day) and a longer duration (≥ 6 months) (Kennel et al. 2010), while the treatment window for SSNHL is narrow (the optimal intervention period is within 72 h). Short‐term supplementation may not be able to promptly repair cochlear damage caused by long‐term Vit D deficiency. Second, the repair of cochlear damage requires Vit D‐mediated gene expression regulation (e.g., genes related to the survival of spiral ganglion neurons) (Zhang et al. 2022), a process that takes weeks to months. However, this study only followed up for 3 months, which may have failed to capture the long‐term efficacy.

### 4.5 Limitations

This study had several key limitations. First, the single‐center design and hospital‐based controls may introduce selection bias, limiting external validity. Second, subgroup sample sizes were small (e.g., recurrent SSNHL: *n* = 10; patients ≥ 60 years: *n* = 10), reducing statistical power for stratified analyses. Third, unmeasured confounding factors (e.g., sun exposure, dietary Vit D intake, VDR gene polymorphisms) may have influenced results. Fourth, the Vit D supplementation dose (800 IU D2 + 250 IU D3) lacks consensus for SSNHL, and the 3‐month follow‐up failed to capture long‐term effects on recurrence. Finally, hearing assessment relied solely on pure‐tone audiometry, without objective indicators (e.g., auditory brainstem response) to minimize subjective bias.

## 5 Conclusion

In summary, this study demonstrates that Vit D deficiency is significantly associated with both the initial onset and recurrence of SSNHL, with the strongest association observed in patients under 30 years old. These findings position Vit D status as a modifiable risk factor worthy of clinical attention in SSNHL management. However, our results indicate that short‐term Vit D supplementation (3 months) following SSNHL diagnosis does not improve hearing recovery outcomes. This discrepancy suggests that while maintaining adequate Vit D levels may have preventive value, therapeutic administration after symptom onset appears insufficient to alter the disease course. Future research should prioritize longitudinal studies to evaluate whether long‐term Vit D maintenance can reduce SSNHL incidence and recurrence, particularly in high‐risk groups such as young adults with Vit D deficiency.

## Author Contributions

Conceptualization: Qi Hui, and Fan Yunping. Methodology: Qi Hui, Tu Xiang, and Xiong Qinglan. Software: Qi Hui. Validation: Qi Hui, Tu Xiang, and Xiong Qinglan. Formal analysis: Qi Hui, and Tu Xiang. Investigation: Yang Juntao, and Xiong Qinglan. Resources: Xiong Qin, and Liu Hongyin. Data curation: Qi Hui, Tu Xiang, and Xiong Qinglan. Writing – original draft preparation: Qi Hui, Tu Xiang, Xiong Qinglan, and Yang Juntao. Writing – review and editing: Chi Mengshi, Yang Shuowei, and Fan Yunping. Visualization: Qi Hui. Supervision: Chi Mengshi, Yang Shuowei, and Fan Yunping. Project administration: Chi Mengshi, Yang Shuowei, and Fan Yunping. Funding acquisition: Fan Yunping. All authors have read and agreed to the published version of the manuscript.

## Funding

The authors have nothing to report.

## Ethics Statement

The study was conducted in accordance with the Declaration of Helsinki and approved by the Ethics Committees of The Seventh Affiliated Hospital of Sun Yat‐sen University (protocol code KY AF/SC‐07/01.0, March 2021). Informed consent was obtained from all subjects involved in the study.

## Data Availability

Data available on request from the authors (The data that support the findings of this study are available from the corresponding author upon reasonable request).
